# Characterization and epidemiologic analysis of mycoplasmal pneumonia of sheep in Qinghai Province

**DOI:** 10.1371/journal.pone.0299928

**Published:** 2024-05-21

**Authors:** Haoyue Yang, Yiming Chen, Siddiq Ur Rahman, Yunpeng Wang, Silu Ni, Yuecai Jiang, Fang Zhu, Dengliang Li, Qihang Cao, Jianjun Chang, Ying Wen, Dekun Chen, Ma Wentao

**Affiliations:** 1 Veterinary Immunology Laboratory, College of Veterinary Medicine, Northwest Agriculture and Forestry University, Yangling, Shaanxi Province, China; 2 Department of Computer Science and Bioinformatics, Khushal Khan Khattak University, Karak, Khyber Pakhtunkhwa, Pakistan; 3 State Key Laboratory of Plateau Ecology and Agriculture, Qinghai University, Xining, Qinghai Province, China; 4 College of Agriculture and Animal Husbandry, Qinghai University, Xining, Qinghai Province, China; Beni Suef University Faculty of Veterinary Medicine, EGYPT

## Abstract

Mycoplasmal pneumonia in sheep and goats usually result covert but huge economic losses in the sheep and goat industry. The disease is prevalent in various countries in Africa and Asia. Clinical manifestations in affected animals include anorexia, fever, and respiratory symptoms such as dyspnea, polypnea, cough, and nasal discharge. Due to similarities with other respiratory infections, accurate diagnosis can be challenging, and isolating the causative organism is often problematic. However, the utilization of molecular techniques, such as PCR, allows for rapid and specific identification of pathogens. Thus, a goat infection model with *Mycoplasma* was established and the pathogen was tested using PCR. The results indicated that this approach could be effectively utilized for the rapid detection of *mycoplasma* in clinical settings. Additionally, the prevalence of contagious pleuropneumonia of sheep in Qinghai Province was further investigated through PCR analysis. A total of 340 nasal swabs were collected from 17 sheep farms in Qinghai province. Among these samples, 84 tested positive for *Mycoplasma mycoides subsp*. *capri* (*Mmc*) and 148 tested positive for *Mycoplasma ovipneumoniae* (*Movi*), resulting in positive rates of 24.71% and 43.53% respectively. Furthermore, our investigation revealed positive PCR results for nasal swabs, trachea, and lung samples obtained from sheep exhibiting symptoms suggestive of *mycoplasma* infection. Moreover, three distinct strains were isolated from these positive samples. Additionally, the inflammatory cytokines of peripheral blood mononuclear cells (PBMCs) were assessed using RT-PCR. The findings demonstrated a high susceptibility of sheep to *Movi* in Qinghai province, with infected sheep displaying an inflammatory response. Consequently, the outcomes of this study will furnish valuable epidemiological insights for the effective prevention and control of this disease within Qinghai Province.

## 1. Introduction

Contagious pleuropneumonia, a widespread disease affecting sheep and goats globally, is caused by pathogens such as *Mycoplasma capricolum subsp*. *capripneumoniae* (*Mccp*), *Mmc*, and *Movi*. [1–3]. Among these, *Mccp* mainly infect ungulates including sheep, goats, and wild animals [4–6], and it is associated with 60% mortality and 90% morbidity in goats [7]. The incubation period of the disease is approximately 45 days. In goats, symptoms include anorexia, fever, and respiratory manifestations such as dyspnea, polypnea, coughing, and nasal discharge [8]. After sheep become infected with *Movi*, the resulting clinical symptoms of the disease bear resemblance to those of *Mccp* infection, characterized by prototypical chronic interstitial pneumonia with significant morbidity and mortality [9]. *Movi* was similar to *Mccp*, coinfecting with common respiratory pathogens [10]. The disease severely impacts both the growth and productivity of lambs [11]. Previous reports have identified respiratory disease as the fifth most significant challenge in domestic sheep production within the United States [12]. Contagious pleuropneumonia caused by *Mccp*, *Mmc* or *Movi* can lead high mortality rates in goats and sheep, resulting in serious consequences [13]. Similarly, they have become increasingly widespread in China. Provinces or districts including Shaanxi, Yunnan, Sichuan, Guizhou, Chongqing, Gansu, Qinghai, Xinjiang and Ningxia have reported its occurrence. The disease has caused significant economic losses in the sheep-farming industry [8, 14–18].

Qinghai Province, being a significant sheep farming region in China, plays a crucial role in the local economy. In recent times, the prevalence of contagious pleuropneumonia of sheep has been observed in various areas of the province, such as Yushu Prefecture, Haiyan County, Guinan County, Pingan County, Huangyuan County, and other counties [19]. However, the reports of this disease in Qinghai Province are sporadic. Hence, it is imperative to gain a comprehensive understanding of the current epidemiological situation of contagious pleuropneumonia in Qinghai. Chu et al. [15] previously reported they used a positive indirect hemagglutination test kit to detect antibodies against contagious pleuropneumonia (including *Movi*, *Mmc* and *Mccp*) in Qinghai Province for epidemiological investigation, but antigen testing is slightly less sensitive and precise than PCR testing [20].

The primary aim of this study is to conduct a comprehensive examination of the prevalence of contagious pleuropneumonia in order to facilitate the timely implementation of targeted control and prevention strategies within Qinghai Province. Thus, we performed PCR analysis (including *Movi*, *Mmc* and *Mccp*) of nasal swabs of sheep with suspected contagious pleuropneumonia in different sheep farms of Qinghai province. The findings not only contribute to our understanding of the prevalence of contagious pleuropneumonia, but also offer valuable insights for the prevention of this disease in Qinghai Province.

## 2. Materials and methods

### 2.1 Bacterial strain and animals

*M599* was isolated from a goat farm, and stored in veterinary immunology laboratory (college of veterinary medicine, Northwest A&F University). Three-month-old female goats were purchased locally and housed at the animal center. All goats exhibited no clinical manifestations of disease and were confirmed to be negative for *Mccp* infection through the utilization of a *Mccp* detection ELISA kit (Abbexa Ltd, Cambridge, UK). At the end of the protocol, the ritualistic slaughter of sheep was conducted in accordance with the methodologies documented in prior scholarly works [21–23]. In brief, the sheep were administered i.v. pentobarbital (30 mg/kg) for anesthesia. After achieving a state of deep anesthesia, followed by i.v. injection of potassium chloride (2 mmol/kg) for euthanasia. The animal experiments were followed by guidelines form Laboratory Animals of the Ministry of Health of China. The study was approved by the Research Ethics Committee of Northwest A&F University. All sample collection was undertaken in accordance with the protocols of the Research Ethics Committee of Northwest A&F University.

### 2.2 Artificial infection of *mycoplasma pneumoniae* in goats

In order to rapidly detect *Mycoplasma* in clinical practice, we established a goat infection model with *mycoplasma*. Culturing methods for lab-isolated strain *M599* was the same as reported in our previous study [8]. Adjust the concentration of *M599* to CCU = 10^9^. The suspension was prepared by diluting the substance in 5 mL of PBS. *M599* was infused into the trachea of goats once a day for a week.

### 2.3 Sample collection in Qinghai province

From March to July 2022, 340 nasal swabs were collected from 17 sheep pastures in Qinghai province by randomized stratified sampling. We recorded the age and region of each swab. None of the sheep had been vaccinated against *Mycoplasma* vaccines. Nasal swabs were composite swabs with the right and left nares, and they were immersed in 3  mL of MEM-KM2 medium. They were kept at 4°C until DNA extraction.

### 2.4 PCR amplification and the evolutionary tree construction

Swabs were eluted by vigorous agitation in 3 mL of MEM-KM2 medium, centrifuged at 3,500 rpm for 5 min, and the supernatant was collected. 200 μL of the supernatant was taken from each sample and lysis-buffer was added to 1 mL respectively. Then they were mixed well, and incubated for 10 min at room temperature. After that, 1 mL of isopropanol solution was added, mixed well, and left for 15 min at -20°C in a freezer. Next, they were centrifuged at 12,000 rpm for 20 min, and the supernatant was discarded. The PCR reaction system was the same as we reported before [8]. The annealing temperatures are 62°C, 47°C, and 55°C, respectively for *Mmc*, *Mccp* and *Movi*. Reaction products were analyzed by 2.0% agarose gel electrophoresis. The PCR products were sequenced in Sangon Biotech (Sangon, Xian, China). The gene sequences were compared with other *mycoplasma* 16S rRNA gene sequences complete gene sequences by BLAST programs in the GenBank database. The *mycoplasma* 16S rRNA gene sequences were aligned using MEGA 7.0 software, where 1000 bootstrap replicates were used to determine the nucleotide sequence distance. Table 1 details the primers used in this study.

**Table 1:** Detailed information for the primers used listed.

Organisms	Amplicon size (bp)	5’- sequence -3’	References
*Mmc*	548	5-CGAAAGCGGCTTACTGGCTTGTT-3	[24] Bascunana *et al*. 1994
		5-TTGAGATTAGCTCCCCTTCACAG-3	
*Mccp*	316	5-ATCATTTTTAATCCCTTCAAG-3	[25] Woubit *et al*. 2004
		5-TACTATGAGTAATTATAATATATGCAA-3	
*Movi*	361	5-TGAACGGAATATGTTAGCTT-3	[13] Mc Auliffe *et al*. 2003
		5-GACTTCATCCTGCACTCTGT-3	

### 2.5 Isolation of peripheral blood mononuclear cells (PBMCs) of sheep in Qinghai province

Draw 2 mL of blood from the jugular vein in goat. The isolation of PBMCs was performed according to our previous reports [8]. Briefly, 2 mL of EDTA treated peripheral blood was centrifuged at 800 g for 5 min, and the supernatant was discarded. The cell pellets were resuspended in 10 mL of ACK Lysis Buffer (Beyotime, Beijing, China) for 10 min at 4°C. Then, the pellets were centrifuged at 800 g for 5 min, and the supernatant was discarded. Next, they were washed with PBS. After that, the cell pellets were resuspended in 40% Percoll solution (GE Healthcare, Little Chalfont, United Kingdom), and centrifuged at 800 g for 10 min. The pellets were collected as PBMCs. Finally, the PBMCs were resuspended with RNAiso (Takara Bio) at -80°C until RNA extraction.

### 2.6 The cytokine levels in goat PBMCs were determined using real-time PCR

Total RNA was isolated using RNAiso Plus (Takara), and reverse transcription was used with a PrimeScript RT Reagent Kit (TIANGEN Biotech, Beijing, China). A SYBR Premix Ex TaqTM II kit (TIANGEN) was used for real-time PCR. The reaction system was carried out according to the instructions. Data were collected using a BioRad CFX Real-Time PCR System (Bio-Rad Laboratories, Inc). PCR was performed using the following conditions: 95°C for 15 s, followed by 40 cycles of 95°C for 10 s, 60°C for 30 s. Gene expression levels were analyzed using the 2^−ΔΔCt^ method. The primers used in this study are listed in S1 Table.

### 2.7 Necropsy and Hematoxylin and Eosin (H & E) staining

At necropsy, lung tissues and trachea were collected. The fresh lung tissues of artificially infected of *mycoplasma pneumoniae*. Sheep lung specimens without clinical symptoms of mycoplasma were collected in Qinghai province. Next, they were fixed in 4% paraformaldehyde for 2 h, respectively. After that, the tissues were further soaked in fresh 4% formaldehyde for 72 h. Then the tissues were paraffin embedded and cut into 4 μm section slices. Next, the HE staining was performed. The slices were observed under an optical microscope.

### 2.8 Statistical analysis

The data were analyzed using IBM SPSS Statistics. All data were evaluated by one-way ANOVA, and they were presented as the mean ± SEM and representative of at least 2 replicates. If the p-value was less than 0.05, statistical differences were considered significant (*p < 0.05, **p < 0.01, ***p < 0.001).

## 3. Results

### 3.1 Artificial infection of *Mycoplasma pneumoniae* in goats

**Fig 1:**
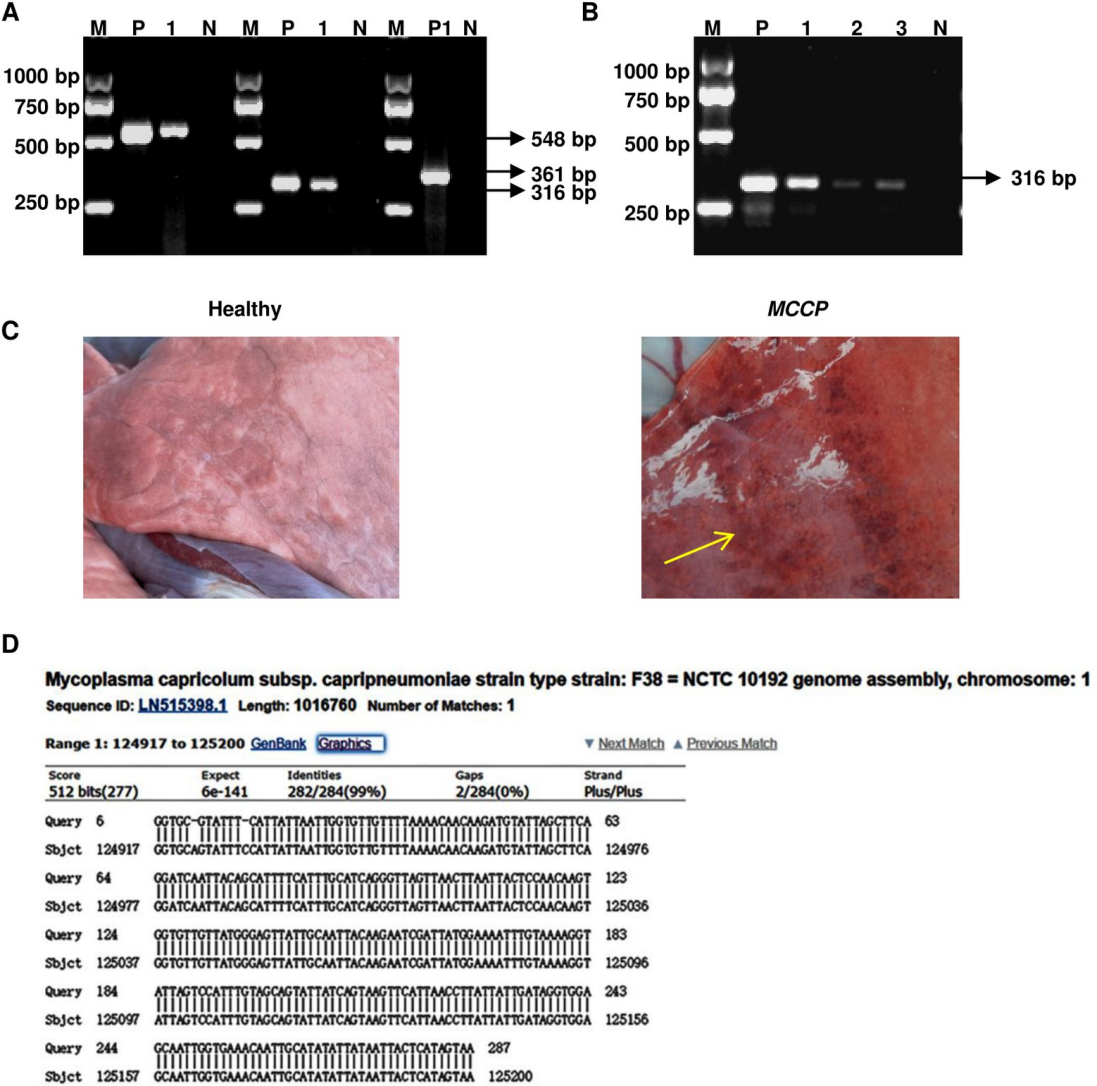
The detection assay was established for *mycoplasma*. (A) Standard strains (*F38*, *Y98*, and *M599*. The lengths of the amplified products of *Mmc*, *Mccp* and *Movi* were 548, 316 and 361 bp, respectively. M: DNA marker, P: positive control (standard strains *F38*), 1: *M599*, P1: positive control (standard strain *Y98*) and N: negative control (double distilled water). (B) PCR-based analysis of DNA samples of infected goats with *M599*. M: DNA marker, P: positive control (*M599*), 1: nose swab, 2: lung homogenates, 3: soaking solution of the trachea, N: negative control (double distilled water). (C) Representative images of the pulmonary tissues of the healthy and infected goats with *M599*. The yellow arrows show hemorrhaging of lung tissue. (D) DNA from infected goat lung tissue was subjected to PCR amplification, and the resulting products were sequenced.

We performed the PCR on samples from artificially infected goats (Fig 1A). Positive results were found in nasal swab, lung homogenate and tracheal soak solution of infected sheep by PCR. And the PCR products of lung tissue were sequenced, which showed that the goats were infected with the *M599* stains (Fig 1B and 1D). At necropsy, pulmonary hemorrhage was observed (Fig 1C). These results suggested that we successfully infected the goats and the PCR method was credible for the detection of *mycoplasma* sheep in clinical setting.

### 3.2 Detection rate of *mycoplasma* infection was determined in Qinghai Province

**Fig 2:**
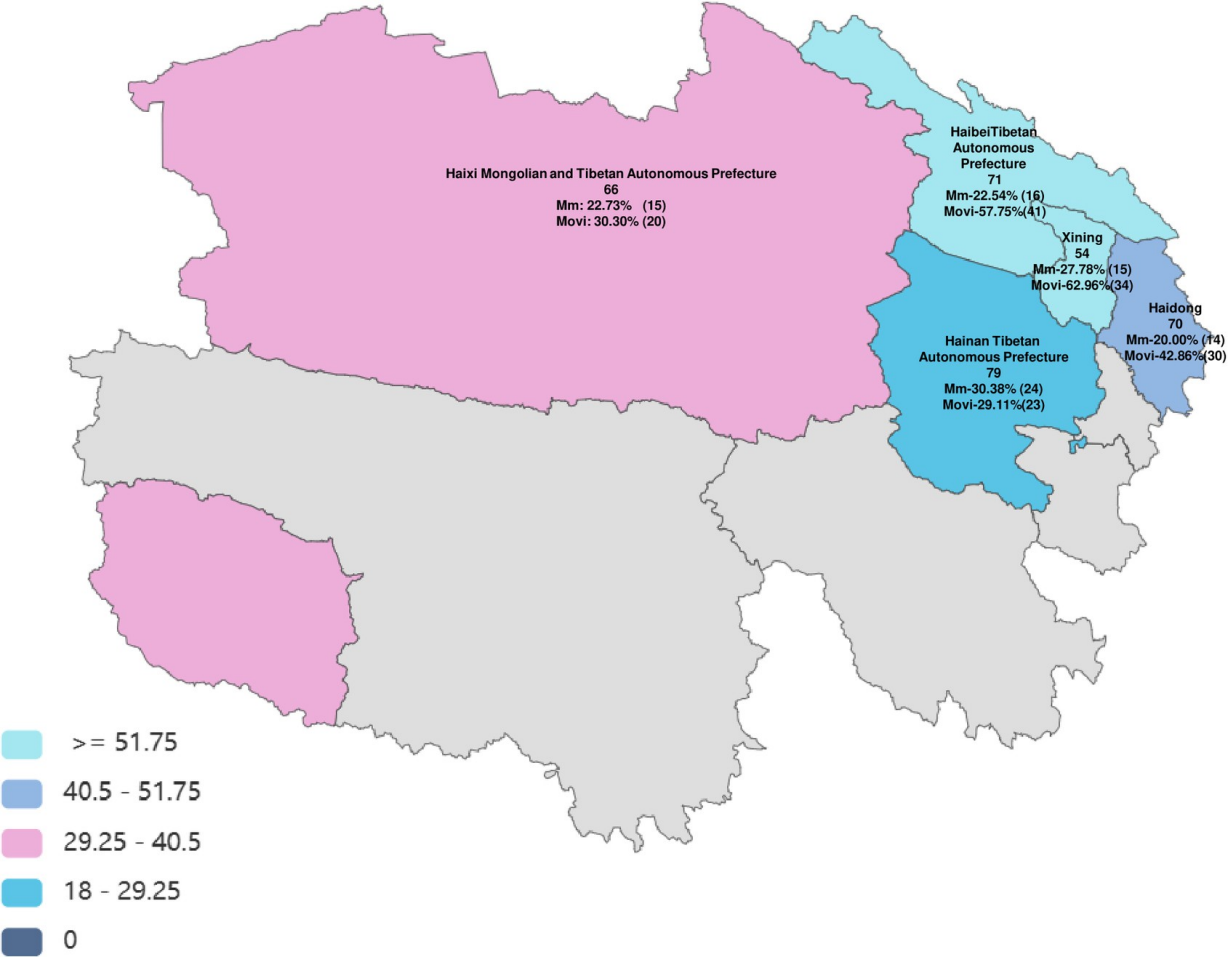
Map of the sample collection sites. The figure illustrates the geographical distribution of collected samples in Qinghai Province and the prevalence rate of *mycoplasma* infection in sheep. This map was drawn on the https://www.ichartcool.com/my.html website. (Disclaimer: The author of this article uploaded the data in the local map and created the final presentation of the map, ensuring that no copyright issues were involved. The basemaps used in the construction of map images were acquired from the Ministry of Natural Resources of the People’s Republic of China. Drawing approval number: GS(2019)3333).

**Fig 3:**
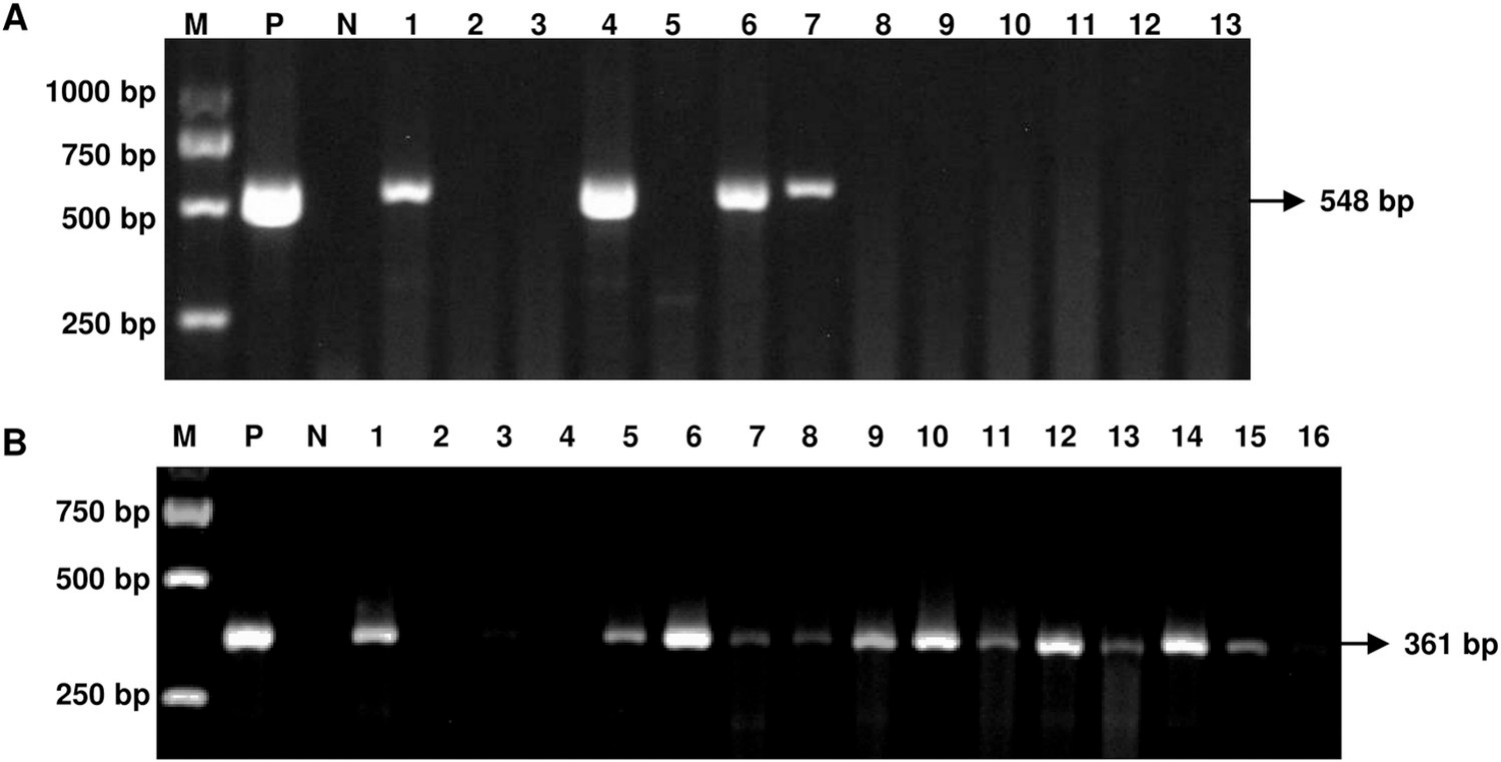
The PCR amplification products of DNA extracted from a partial sheep nose swab were subjected to agarose gel electrophoresis. (A) PCR-based analysis of DNA samples of the nose swab were detected in sheep with *Mmc*. M: DNA marker, P: positive control (standard strains *F38*), N: negative control (double distilled water), 1–13: Nose swab samples. (B) The nose swab samples were detected in sheep with *Movi* by PCR. M: DNA marker, P: positive control (standard strains *Y98*), N: negative control (double distilled water), 1–16: Nose swab samples.

**Fig 4:**
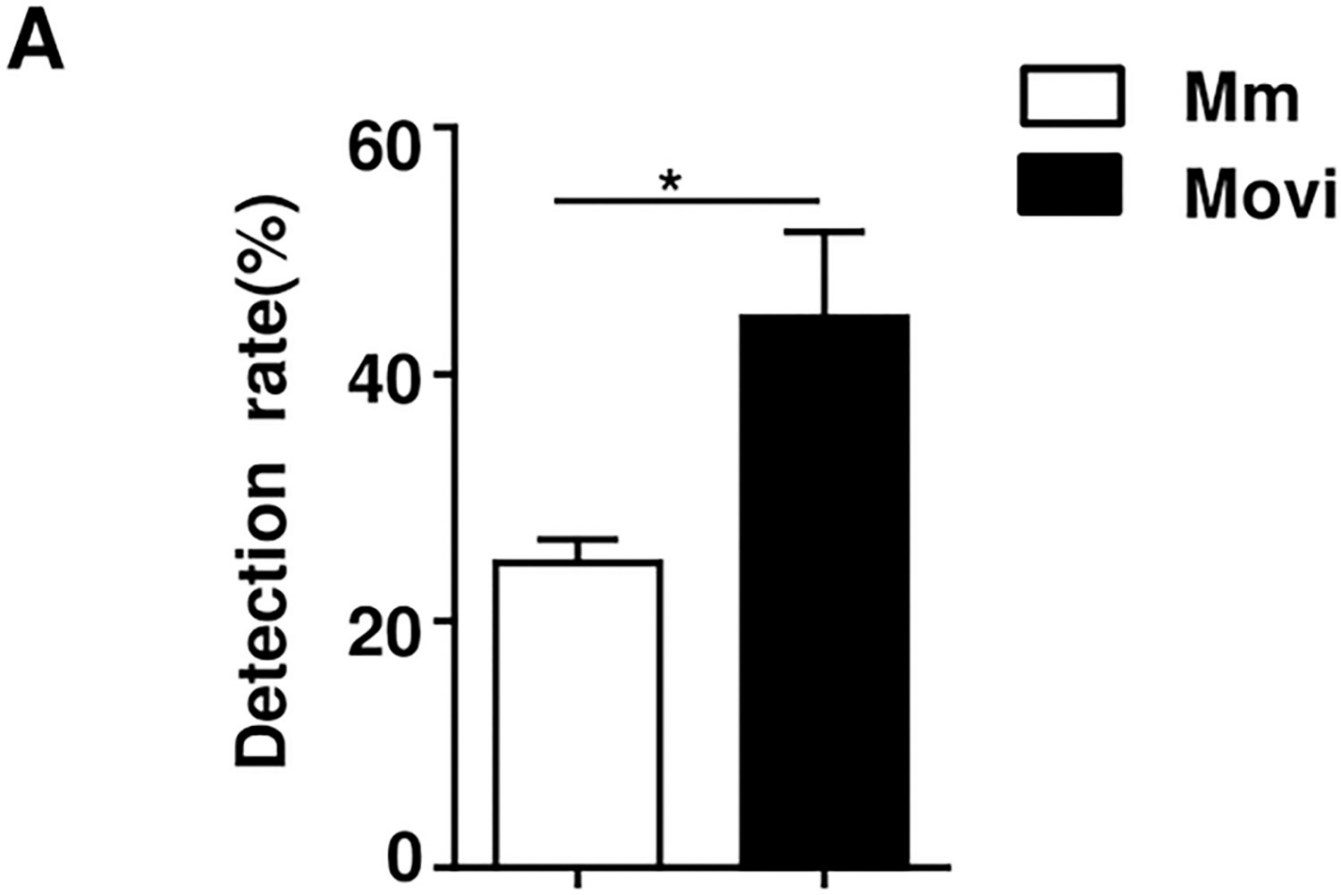
Statistical analysis of PCR positive detection rates of sheep nasal swab DNA in Qinghai Province (includes *Mmc* and *Movi*). * P < 0.05.

**Fig 5:**
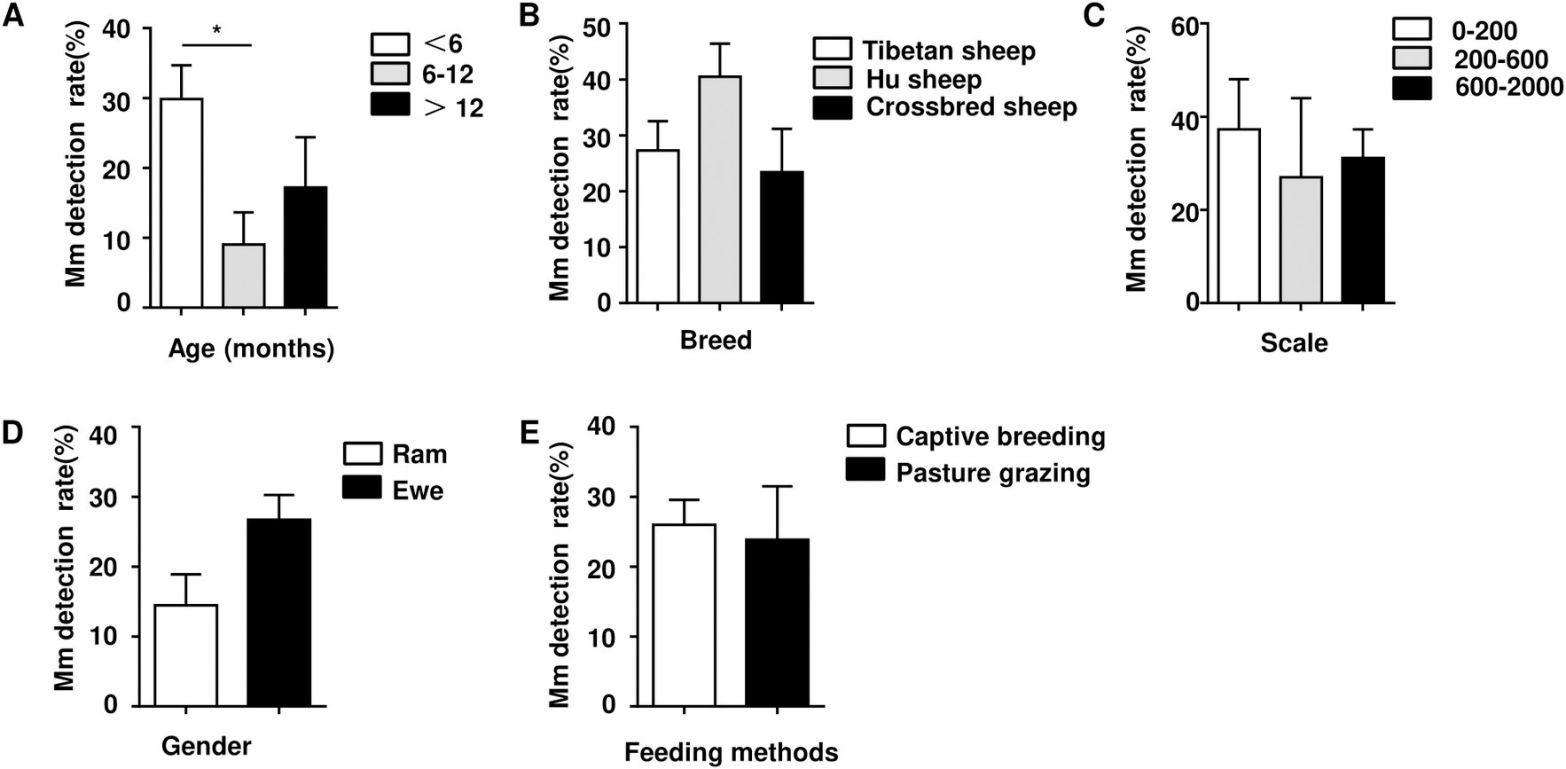
An analysis was conducted on the PCR positive detection rate of *Mmc* in sheep nasal swabs collected from Qinghai Province. (A) Statistical analysis categorized by age. (B) Statistical analysis categorized by breed. (C) Statistical analysis categorized by scale. (D) Statistical analysis categorized by gender. (E) Statistical analysis categorized by feeding methods. * P < 0.05.

**Fig 6:**
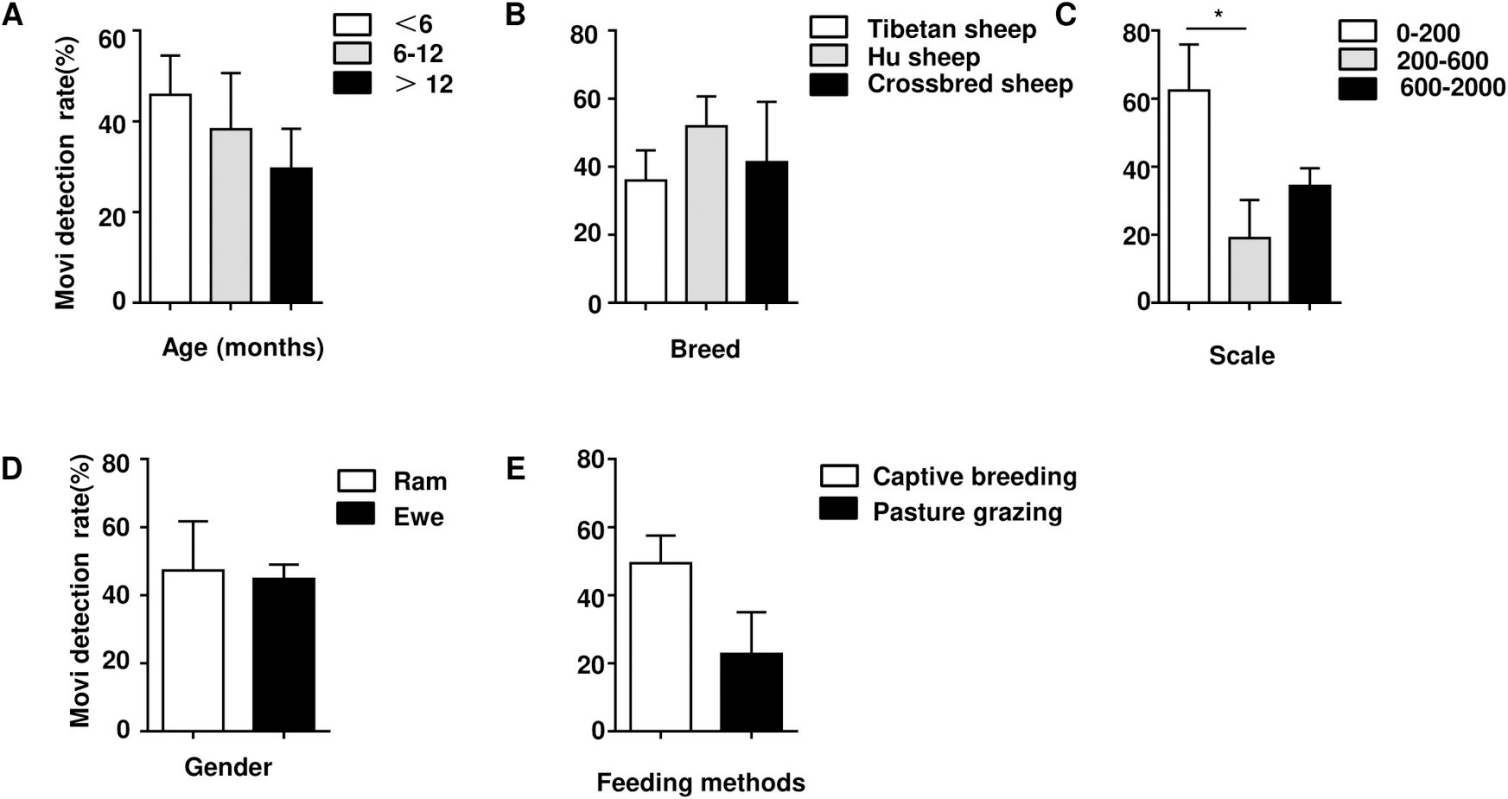
The positive detection rate of *Movi* was performed in Qinghai Province. (A) Statistical analysis of *Movi* was performed as age. (B) Statistical analysis of *Movi* was performed as breed. (C) Statistical analysis of *Movi* was performed as scale. (D) Statistical analysis of *Movi* was performed as gender. (E) Statistical analysis of *Movi* was performed as feeding. * P < 0.05.

The positive detection rate of *Mycoplasma* (include *Mmc* and *Movi*) infection was determined by PCR using samples collected from sheep in Qinghai Province. (As the positive detection rate of *Mccp* is very low, therefore, the data were not shown). In order to make the experimental results more clear, we plotted the sampling geographical location and labeled the positive detection rate (Fig 2). PCR showed that some samples of nasal swab showed positive bands (Fig 3A, 3B and S1 Fig). The statistical analysis demonstrates, among the 340 tested nasal swabs, 84 *Mmc* positives and 148 *Movi* positives were detected. The positive rates were 24.71% and 43.53%, respectively. And the positive rate of *Movi* was statistically significantly higher than that of *Mmc* (Fig 4A). For *Mmc*, the positive rate of sheep aged 0–6 months was statistically significantly higher than that of sheep aged 6–12 months (Fig 5A). Compared with the rams, the positive rate of *Mmc* in ewes was 0.6-fold higher than them, although the difference did not reach significance (Fig 5D). For other information (including breed, scale, and feeding methods), there were no significant differences (Fig 5B, 5C and 5E). For *Movi*, the detection rate was highest in sheep aged 0–6 months, although the difference didn’t reach significance (Fig 6A). The positive rate of *Movi* detection in farms with scale lower than 200 sheep was statistically significantly higher than others (Fig 6C). For the rearing condition, the detection rate of *Movi* also showed a trend to decrease in the grazing regimen, although it didn’t reach statistical significance (Fig 6E). For the other information (including breed and gender), there were no significant differences (Fig 6B and 6D). The positive rates of *Mycoplasma* of nasal swabs were listed by locations, gender, breed, scale, feeding methods and age (S2 Table).

### 3.3 Clinical manifestations and pathological damage of sheep mycoplasmal pneumonia in Qinghai Province

**Fig 7:**
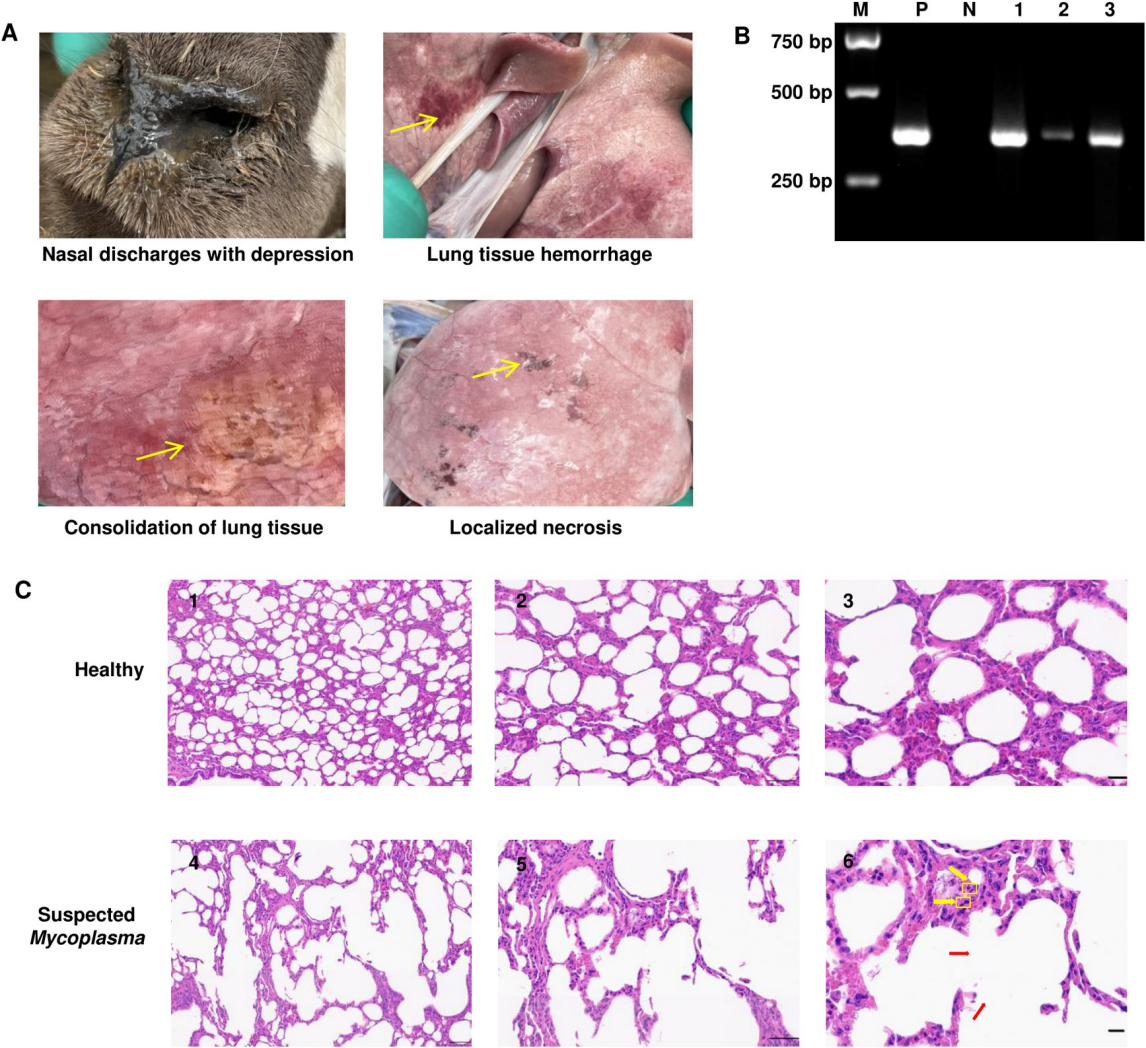
Clinical symptoms and pathological lesion of sheep infected *mycoplasma* in Qinghai province. (A) The clinical manifestation is the nose with nasal fluid and mental depression. The pathological changes are lung tissue hemorrhage, consolidation and localized necrosis. (B) Specificity of the PCR for bacterial cultures, M: DNA marker, P: positive control (standard strains *Y98*), N: negative control (double distilled water), 1: nose swab, 2: lung homogenates, 3: soaking solution of the trachea. (C) Lung histology sections. The yellow arrows show neutrophil infiltration, and the red arrow shows distension of alveoli, alveolar rupture and fusion forming pulmonary bullae.

Typical symptoms include nasal discharge, a high fever (42.6°C) and poor appetite in clinically infected sheep with *Mycoplasma*. After dissection, we found that the lung tissues showed pathological changes, including hemorrhage, consolidation, and localized necrosis (Fig 7A). After verification using PCR, we found that the nose swab, lung homogenates and soaking solution of the trachea were positive in the infected sheep (Fig 7B). Microscopically, at high magnification, the normal lung tissue had a much more homogeneous histology, and the alveolar walls were thin. Compared to healthy controls, the infected lung tissues were infiltrated with inflammatory cells and loss their alveolar walls (Fig 7C). In line with these findings, the expression levels of several inflammatory cytokines, such as TNF-α and IFN-γ increased significantly in diseased goats as compared to that of the healthy ones (S2 Fig).

### 3.4 Pathogen identification and genetic characterization of *M*. *ovipneumoniae*

**Fig 8:**
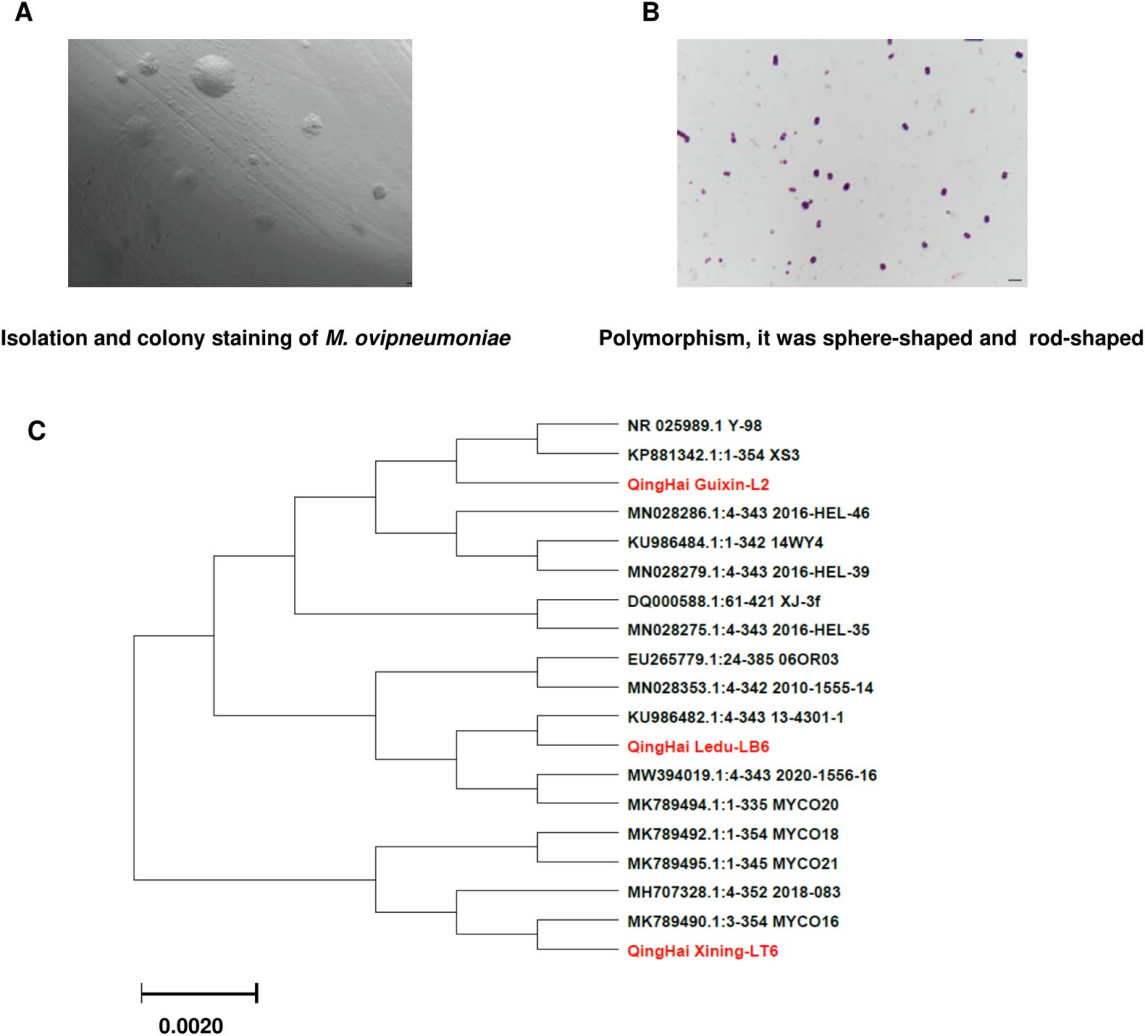
Pathogen and genetic characterization were identified as *M*. *ovipneumoniae*. (A) Colony morphology of *M*.*ovipneumoniae* (40×). (B) It was polymorphous, sphere-shaped and rod-shaped (100×). (C) Phylogenetic tree of *M*. *ovipneumoniae*. MEGA 7.0 was employed to construct neighbor-joining trees.

Three strains were isolated from the positive samples of sheep nasal swabs collected. The strains grew well on the MEM-KM2 medium. After 7–9 days, the white transparent spots of needle tip size were visualized on solid medium, and the colonies were visualized in a low-power microscopic field and their sizes varied (Fig 8A). The bacterial precipitate collected by centrifugation is dyed into blue purple particles by Giemsa staining (Fig 8B). The software MEGA 7.0 was used to reconstruct the evolutionary tree based on partial sequences of the *M*. *ovipneumoniae* 16S rRNA gene amplified with specific primers. The isotypes labeled in red were isolated in the study. According to the sequence alignment results, the isolates were clustered in the same branch with the correlating strain of *M*. *ovipneumoniae* (Fig 8C).

## 4. Discussion

Both sheep numbers and breeds of China are leading in the world, and sheep farming is important for the economies [26]. In 2020, the number of goats reached 133.4525 million, and the amount of sheep’s depository reached 1730.953 million in China (Data source: www.AskCl.com). Qinghai province contains significant amounts of pasture, and it is one of the five largest animal pastoral areas in China [27]. The sheep industry significantly contributes to the economic development of Qinghai province, as evidenced by the increasing volume of sheep’s depository and outlet, resulting in substantial economic benefits. However, the prevalence of respiratory disease poses a significant threat to the sheep industry, leading to considerable economic losses. Among these respiratory diseases, *Mycoplasma pneumonia* stands out as one of the most crucial infectious diseases that jeopardize the production of sheep and goats. This respiratory ailment has been reported in numerous countries, causing substantial economic damage [16, 28]. One of the diseases that contributes to significant economic losses in endemic regions is contagious caprine pleuropneumonia (CCPP), amounting to approximately US$507 million [17]. Additionally, the presence of *M*. *ovipneumoniae* infection, even in asymptomatic carriers, has been found to impede the growth rates of lambs [29]. This disease is prevalent across various provinces in China and exhibits a tendency to expand. According to relevant reports, which are mainly case descriptions, the incidence and mortality rate of *mycoplasma pneumonia* in sheep or goats have also shown an upward trend in high-cold areas.

This study represents the first investigation into the epidemiology of mycoplasmal pneumonia in sheep within Qinghai province. In the initial phase, we performed artificial infection tests on goats to examine the presence of mycoplasma pneumoniae through PCR analysis of nasal swabs and lung samples. In the current study, our objective was to assess the prevalence of mycoplasma pneumoniae infection in Qinghai Province by employing PCR analysis on nasal swab samples collected from five distinct regions. The findings revealed an average positive rate exceeding 24%, indicating a high prevalence of mycoplasma pneumoniae infection in Qinghai Province.

The average positive rate of *M*. *ovipneumoniae* by PCR in this study was 43.53%, which was higher than that found in Xinjiang (*M*. *ovipneumoniae* nasal swabs average positive rate was 10.18%), and the overall nasal swab positive rate for *M*. *ovipneumoniae* was 40.78% [9, 16], whereas lower than the results reported by Xiu-lan, X et al. (the average positive rate was 74%) in Ningxia of China [30]. Rong, G et al reported the positive rate of goat serum specimen pneumoniacum was 31.7% in Hainan [31], and Guo, H reported the positive rate of sheep serum was 30.4% in Qinghai Province [15]. So, even within the same country, variability may exist in different regions. In Qinghai, the detection rate of mycoplasma pneumonia has shown an increase when compared to data from a decade ago, suggesting a continuous rise in bacterial infection rates. Our survey findings indicate a high detection rate of both *Mmc* and *Movi* in lambs, with average positive rates of 29.85% and 45.82% respectively, aligning with previous reports. In comparison, *M*. *ovipneumoniae* emerges as the primary infectious agent in sheep [11, 16, 32]. In comparison, *M*. *ovipneumoniae* emerges as the primary infectious agent in sheep. Our results show that the Hu sheep has a higher average positive rate (*Mmc*-32.39%, *Movi*-41.51%) compared to the other two breeds, which is consistent with a previous report [16]. This indicates that sheep breeds have different susceptibilities to *M*. *ovipneumoniae*. Alternatively, the average positive rate of *Mmc* and *Movi* was 26.01% and 49.4%, respectively in captivity, which is higher than sheep-grazing (the detection rate was 19.09% and 22.74%, respectively). The higher prevalence of *mycoplasma pneumonia* in captive goats can be attributed to the more crowded conditions they are typically kept in, which serves as a predisposing factor. Additionally, our findings indicate that the rate of detecting the disease in free-ranging sheep (small scale) is greater than in intensively farmed sheep, potentially due to the farmers’ professional knowledge and expertise in managing the farming process. Generally, the incidence of *mycoplasma pneumonia* in sheep is influenced by various factors such as disease outbreaks, breed, location, environment, age, and stress levels. Furthermore, the detection rates of the disease have been observed to correlate with the sample size of studies or the number of affected cases. Based on the findings, it can be inferred that the susceptibility of sheep in Qinghai Province to mycoplasma may be influenced by factors such as age, gender, and rearing condition.

## Supporting information

S1 Fig Gel electrophoresis of PCR products of DNA amplification from a sheep nose swab in Qinghai province, shown as part of the sample. (TIF)

S2 Fig The expression of inflammatory cytokines examined in PBMCs. (TIF)

S1 Table The primers used in this study for RT-PCR. (DOCX)

S2 Table Positive detection rate of mycoplasma infection was determined by PCR. (DOCX)

S1 Raw images (ZIP)

## References

[1] Carmichael LE, St GT, Sullivan ND, Horsfall N. Isolation, propagation, and characterization studies of an ovine mycoplasma responsible for proliferative interstitial pneumonia. Cornell Vet. 1972 1972 Oct;62(4):654–79. Available from: http://www.ncbi.nlm.nih.gov/entrez/query.fcgi?cmd=Retrieve&db=pubmed&dopt=Abstract&list_uids=4672899&query_hl=1 4672899

[2] Kusiluka LJ, Ojeniyi B, Friis NF, Kazwala RR, Kokotovic B. Mycoplasmas isolated from the respiratory tract of cattle and goats in tanzania. Acta Vet Scand. 2000 2000;41(3):299–309. Available from: http://www.ncbi.nlm.nih.gov/entrez/query.fcgi?cmd=Retrieve&db=pubmed&dopt=Abstract&list_uids=11126579&query_hl=1 doi: 10.1186/BF03549638 11126579 PMC7996426

[3] March JB, Gammack C, Nicholas R. Rapid detection of contagious caprine pleuropneumonia using a mycoplasma capricolum subsp. Capripneumoniae capsular polysaccharide-specific antigen detection latex agglutination test. J Clin Microbiol. 2000 2000 Nov;38(11):4152–59. Available from: http://www.ncbi.nlm.nih.gov/entrez/query.fcgi?cmd=Retrieve&db=pubmed&dopt=Abstract&list_uids=11060083&query_hl=1 doi: 10.1128/JCM.38.11.4152-4159.2000 PMC87556 11060083

[4] Arif A, Schulz J, Thiaucourt F, Taha A, Hammer S. Contagious caprine pleuropneumonia outbreak in captive wild ungulates at al wabra wildlife preservation, state of qatar. J Zoo Wildl Med. 2007 2007 Mar;38(1):93–96. Available from: http://www.ncbi.nlm.nih.gov/entrez/query.fcgi?cmd=Retrieve&db=pubmed&dopt=Abstract&list_uids=17469281&query_hl=1 doi: 10.1638/05-097.1 17469281

[5] Nicholas R., Ayling R., McAuliffe L. Mycoplasma diseases of ruminants. Cabi, Wallingford. 2008.

[6] Cetinkaya B, Kalin R, Karahan M, Atil E, Manso-Silvan L, Thiaucourt F. Detection of contagious caprine pleuropneumonia in east turkey. Rev Sci Tech. 2009 2009 Dec;28(3):1037–44. Available from: http://www.ncbi.nlm.nih.gov/entrez/query.fcgi?cmd=Retrieve&db=pubmed&dopt=Abstract&list_uids=20462161&query_hl=1 doi: 10.20506/rst.28.3.1944 20462161

[7] Yu Z, Wang T, Sun H, Xia Z, Zhang K, Chu D, et al. Contagious caprine pleuropneumonia in endangered tibetan antelope, china, 2012. Emerg Infect Dis. 2013 2013 Dec;19(12):2051–53. Available from: http://www.ncbi.nlm.nih.gov/entrez/query.fcgi?cmd=Retrieve&db=pubmed&dopt=Abstract&list_uids=24274020&query_hl=1 doi: 10.3201/eid1912.130067 PMC3840868 24274020

[8] Ma WT, Gu K, Yang R, Tang XD, Qi YX, Liu MJ, et al. Interleukin-17 mediates lung injury by promoting neutrophil accumulation during the development of contagious caprine pleuropneumonia. Vet Microbiol. 2020 2020 Apr;243:108651. Available from: http://www.ncbi.nlm.nih.gov/entrez/query.fcgi?cmd=Retrieve&db=pubmed&dopt=Abstract&list_uids=32273025&query_hl=1 doi: 10.1016/j.vetmic.2020.108651 32273025

[9] Zhao JY, Du YZ, Song YP, Zhou P, Chu YF, Wu JY. Investigation of the prevalence of mycoplasma ovipneumoniae in southern xinjiang, china. J Vet Res. 2021 2021 Jun;65(2):155–60. Available from: http://www.ncbi.nlm.nih.gov/entrez/query.fcgi?cmd=Retrieve&db=pubmed&dopt=Abstract&list_uids=34250299&query_hl=1 doi: 10.2478/jvetres-2021-0021 34250299 PMC8256467

[10] Chu Y, Yan X, Gao P, Zhao P, He Y, Liu J, et al. Molecular detection of a mixed infection of goatpox virus, orf virus, and mycoplasma capricolum subsp. Capripneumoniae in goats. J Vet Diagn Invest. 2011 2011 Jul;23(4):786–89. Available from: http://www.ncbi.nlm.nih.gov/entrez/query.fcgi?cmd=Retrieve&db=pubmed&dopt=Abstract&list_uids=21908324&query_hl=1 doi: 10.1177/1040638711407883 21908324

[11] Besser TE, Levy J, Ackerman M, Nelson D, Manlove K, Potter KA, et al. A pilot study of the effects of mycoplasma ovipneumoniae exposure on domestic lamb growth and performance. Plos One. 2019 2019;14(2):e0207420. Available from: http://www.ncbi.nlm.nih.gov/entrez/query.fcgi?cmd=Retrieve&db=pubmed&dopt=Abstract&list_uids=30730893&query_hl=1 doi: 10.1371/journal.pone.0207420 30730893 PMC6366759

[12] Manlove K, Branan M, Baker K, Bradway D, Cassirer EF, Marshall KL, et al. Risk factors and productivity losses associated with mycoplasma ovipneumoniae infection in united states domestic sheep operations. Prev Vet Med. 2019 2019 Jul 1;168:30–38. Available from: http://www.ncbi.nlm.nih.gov/entrez/query.fcgi?cmd=Retrieve&db=pubmed&dopt=Abstract&list_uids=31097121&query_hl=1 doi: 10.1016/j.prevetmed.2019.04.006 31097121

[13] McAuliffe L, Hatchell FM, Ayling RD, King AI, Nicholas RA. Detection of mycoplasma ovipneumoniae in pasteurella-vaccinated sheep flocks with respiratory disease in england. Vet Rec. 2003 2003 Nov 29;153(22):687–88. Available from: http://www.ncbi.nlm.nih.gov/entrez/query.fcgi?cmd=Retrieve&db=pubmed&dopt=Abstract&list_uids=14682543&query_hl=1 doi: 10.1136/vr.153.22.687 14682543

[14] Wesonga HO, Bolske G, Thiaucourt F, Wanjohi C, Lindberg R. Experimental contagious caprine pleuropneumonia: a long term study on the course of infection and pathology in a flock of goats infected with mycoplasma capricolum subsp. Capripneumoniae. Acta Vet Scand. 2004 2004;45(3–4):167–79. Available from: http://www.ncbi.nlm.nih.gov/entrez/query.fcgi?cmd=Retrieve&db=pubmed&dopt=Abstract&list_uids=15663077&query_hl=1 doi: 10.1186/1751-0147-45-167 PMC1820987 15663077

[15] Han Guo, Yue-feng Chu, Ping Zhao, Peng-cheng Gao. Serological investigation on *mycoplasma oviovipneumoniae* in qinghai province (in chinese). Animal Husbandry and Feed Science. 2009;4-5(1):34–35.

[16] Cheng C, Jun Q, Qingling M, Zhengxiang H, Yu M, Xuepeng C, et al. Serological and molecular survey of sheep infected with mycoplasma ovipneumoniae in xinjiang, china. Trop Anim Health Prod. 2015 2015 Dec;47(8):1641–47. Available from: http://www.ncbi.nlm.nih.gov/entrez/query.fcgi?cmd=Retrieve&db=pubmed&dopt=Abstract&list_uids=26315151&query_hl=1 doi: 10.1007/s11250-015-0908-2 26315151

[17] Iqbal YM, Raffiq PO, Tauseef BS, Ahmed BR, Gopalakrishnan A, Karthik K, et al. Contagious caprine pleuropneumonia—a comprehensive review. Vet Q. 2019 2019 Dec;39(1):1–25. Available from: http://www.ncbi.nlm.nih.gov/entrez/query.fcgi?cmd=Retrieve&db=pubmed&dopt=Abstract&list_uids=30929577&query_hl=1 doi: 10.1080/01652176.2019.1580826 PMC6830973 30929577

[18] Wang J, Li R, Sun X, Liu L, Hao X, Wang J, et al. Development and validation of the isothermal recombinase polymerase amplification assays for rapid detection of mycoplasma ovipneumoniae in sheep. Bmc Vet Res. 2020 2020 Jun 1;16(1):172. Available from: http://www.ncbi.nlm.nih.gov/entrez/query.fcgi?cmd=Retrieve&db=pubmed&dopt=Abstract&list_uids=32487081&query_hl=1 doi: 10.1186/s12917-020-02387-3 32487081 PMC7268655

[19] Zhuoma RENQing, Yingna Jian, Guanghua Wang, Xiuping Li. Diagnosis of sheep infected with pleuropneumonia in gangcha county (in chinese). Chinese Qinghai Journal of Animal and Veterinary Science. 2022;52(3).

[20] Noda K, Matsuda K, Yagishita S, Maeda K, Akiyama Y, Terada-Hirashima J, et al. A novel highly quantitative and reproducible assay for the detection of anti-SARS-cov-2 igg and igm antibodies. Sci Rep. 2021 2021 Mar 4;11(1):5198. Available from: http://www.ncbi.nlm.nih.gov/entrez/query.fcgi?cmd=Retrieve&db=pubmed&dopt=Abstract&list_uids=33664294&query_hl=1 doi: 10.1038/s41598-021-84387-3 33664294 PMC7933429

[21] Cattaneo GF, Herrmann AM, Eiden SA, Wieser M, Kellner E, Doostkam S, et al. Selective intra-carotid blood cooling in acute ischemic stroke: a safety and feasibility study in an ovine stroke model. J Cereb Blood Flow Metab. 2021 2021 Nov;41(11):3097–110. Available from: http://www.ncbi.nlm.nih.gov/entrez/query.fcgi?cmd=Retrieve&db=pubmed&dopt=Abstract&list_uids=34159825&query_hl=1 doi: 10.1177/0271678X211024952 PMC8756475 34159825

[22] Krebs J, Ferguson SJ, Nuss K, Leskosek B, Hoerstrup SP, Goss BG, et al. Plasma levels of endothelin-1 after a pulmonary embolism of bone marrow fat. Acta Anaesthesiol Scand. 2007 2007 Sep;51(8):1107–14. Available from: http://www.ncbi.nlm.nih.gov/entrez/query.fcgi?cmd=Retrieve&db=pubmed&dopt=Abstract&list_uids=17697307&query_hl=1 doi: 10.1111/j.1399-6576.2007.01369.x 17697307

[23] Han F, Li J, Zhao R, Liu L, Li L, Li Q, et al. Identification and co-expression analysis of long noncoding rnas and mrnas involved in the deposition of intramuscular fat in aohan fine-wool sheep. Bmc Genomics. 2021 2021 Feb 1;22(1):98. Available from: http://www.ncbi.nlm.nih.gov/entrez/query.fcgi?cmd=Retrieve&db=pubmed&dopt=Abstract&list_uids=33526009&query_hl=1 doi: 10.1186/s12864-021-07385-9 33526009 PMC7852088

[24] Bascunana CR, Mattsson JG, Bolske G, Johansson KE. Characterization of the 16s rrna genes from mycoplasma sp. Strain f38 and development of an identification system based on pcr. J Bacteriol. 1994 1994 May;176(9):2577–86. Available from: http://www.ncbi.nlm.nih.gov/entrez/query.fcgi?cmd=Retrieve&db=pubmed&dopt=Abstract&list_uids=8169205&query_hl=1 doi: 10.1128/jb.176.9.2577-2586.1994 8169205 PMC205395

[25] Woubit S, Lorenzon S, Peyraud A, Manso-Silvan L, Thiaucourt F. A specific pcr for the identification of mycoplasma capricolum subsp. Capripneumoniae, the causative agent of contagious caprine pleuropneumonia (ccpp). Vet Microbiol. 2004 2004 Nov 30;104(1–2):125–32. Available from: http://www.ncbi.nlm.nih.gov/entrez/query.fcgi?cmd=Retrieve&db=pubmed&dopt=Abstract&list_uids=15530747&query_hl=1 doi: 10.1016/j.vetmic.2004.08.006 15530747

[26] Jia J, Chen Q, Gui L, Jin J, Li Y, Ru Q, et al. Association of polymorphisms in bone morphogenetic protein receptor-1b gene exon-9 with litter size in dorset, mongolian, and small tail han ewes. Asian-Australas J Anim Sci. 2019 2019 Jul;32(7):949–55. Available from: http://www.ncbi.nlm.nih.gov/entrez/query.fcgi?cmd=Retrieve&db=pubmed&dopt=Abstract&list_uids=30744327&query_hl=1 doi: 10.5713/ajas.18.0541 30744327 PMC6601060

[27] Han R, Yang JF, Mukhtar MU, Chen Z, Niu QL, Lin YQ, et al. Molecular detection of anaplasma infections in ixodid ticks from the qinghai-tibet plateau. Infect Dis Poverty. 2019 2019 Feb 7;8(1):12. Available from: http://www.ncbi.nlm.nih.gov/entrez/query.fcgi?cmd=Retrieve&db=pubmed&dopt=Abstract&list_uids=30728069&query_hl=1 doi: 10.1186/s40249-019-0522-z 30728069 PMC6366118

[28] Rifatbegovic M, Maksimovic Z, Hulaj B. Mycoplasma ovipneumoniae associated with severe respiratory disease in goats. Vet Rec. 2011 2011 May 28;168(21):565. Available from: http://www.ncbi.nlm.nih.gov/entrez/query.fcgi?cmd=Retrieve&db=pubmed&dopt=Abstract&list_uids=21610002&query_hl=1 doi: 10.1136/vr.d886 21610002

[29] Robinson E, Schulein C, Jacobson BT, Jones K, Sago J, Huber V, et al. Pathophysiology of influenza d virus infection in specific-pathogen-free lambs with or without prior mycoplasma ovipneumoniae exposure. Viruses. 2022 2022 Jun 28;14(7). Available from: http://www.ncbi.nlm.nih.gov/entrez/query.fcgi?cmd=Retrieve&db=pubmed&dopt=Abstract&list_uids=35891403&query_hl=1 doi: 10.3390/v14071422 35891403 PMC9321583

[30] Xiu-lan X, Eerhehua, Shuai L, Xiao-ming M, Yu-qiong L, Ying-kang L. The survey of mycoplasma ovipneumoniae in different sheep breeds in ningxia area(in chinese). Chinese Journal of Veterinary Medicine. 2017;53(6).

[31] Rong G, Zhao JM, Hou GY, Zhou HL. Seroprevalence and molecular detection of mycoplasma ovipneumoniae in goats in tropical china. Trop Anim Health Prod. 2014 2014 Dec;46(8):1491–95. Available from: http://www.ncbi.nlm.nih.gov/entrez/query.fcgi?cmd=Retrieve&db=pubmed&dopt=Abstract&list_uids=25099398&query_hl=1 doi: 10.1007/s11250-014-0645-y 25099398

[32] Grossman PC, Schneider DA, Herndon DR, Knowles DP, Highland MA. Differential pulmonary immunopathology of domestic sheep (ovis aries) and bighorn sheep (ovis canadensis) with mycoplasma ovipneumoniae infection: a retrospective study. Comp Immunol Microbiol Infect Dis. 2021 2021 Jun;76:101641. Available from: http://www.ncbi.nlm.nih.gov/entrez/query.fcgi?cmd=Retrieve&db=pubmed&dopt=Abstract&list_uids=33689940&query_hl=1 doi: 10.1016/j.cimid.2021.101641 33689940

