# Supplementary figures and images for: Characterization and epidemiologic analysis of mycoplasmal pneumonia of sheep in Qinghai Province
Source: PLoS One. 2024 May 21;19(5):e0299928. doi: 10.1371/journal.pone.0299928 (PMC11108190; doi:10.1371/journal.pone.0299928)

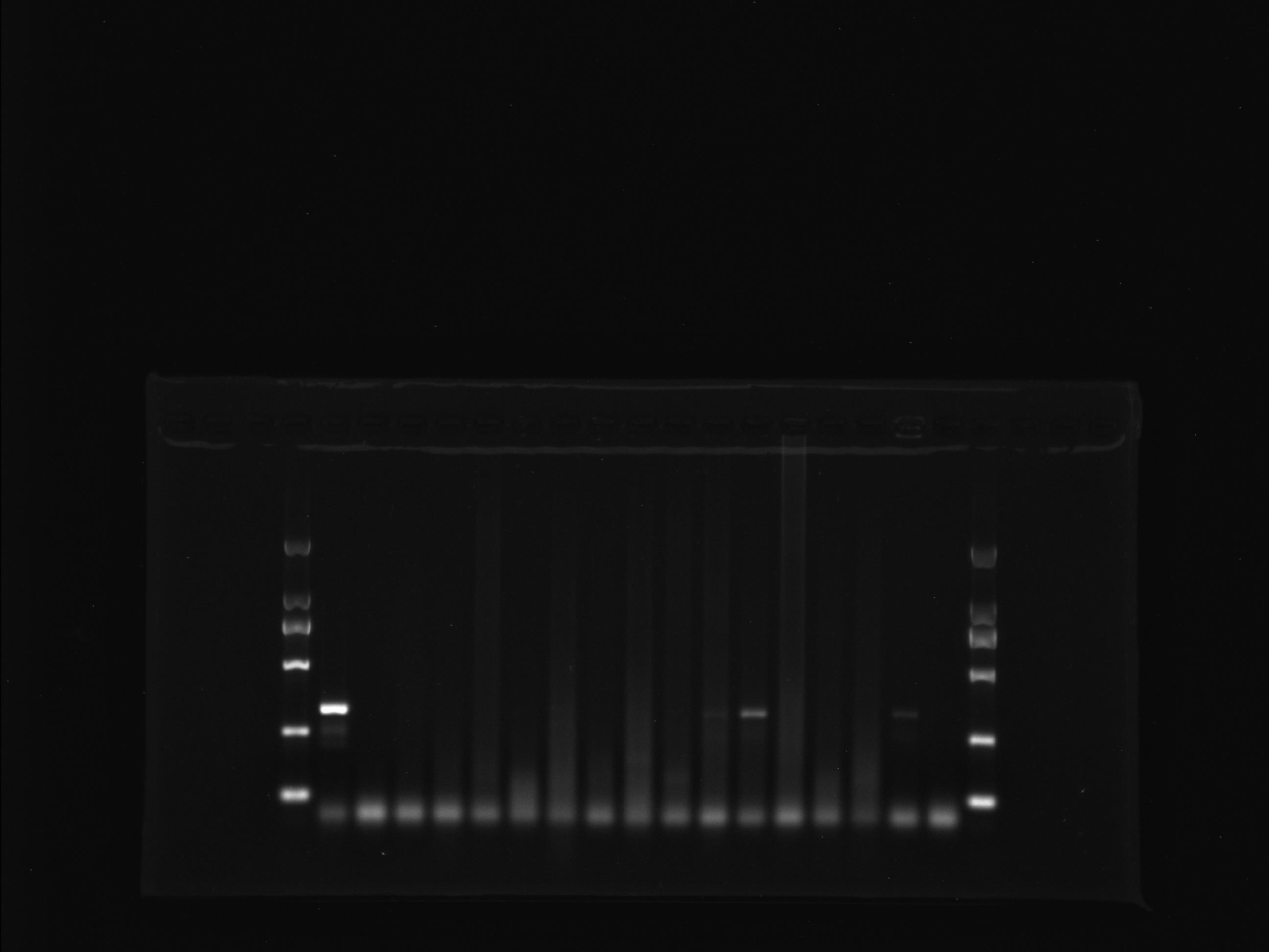

Supplement: S1 Fig — (TIF) [file pone.0299928.s001.tif]

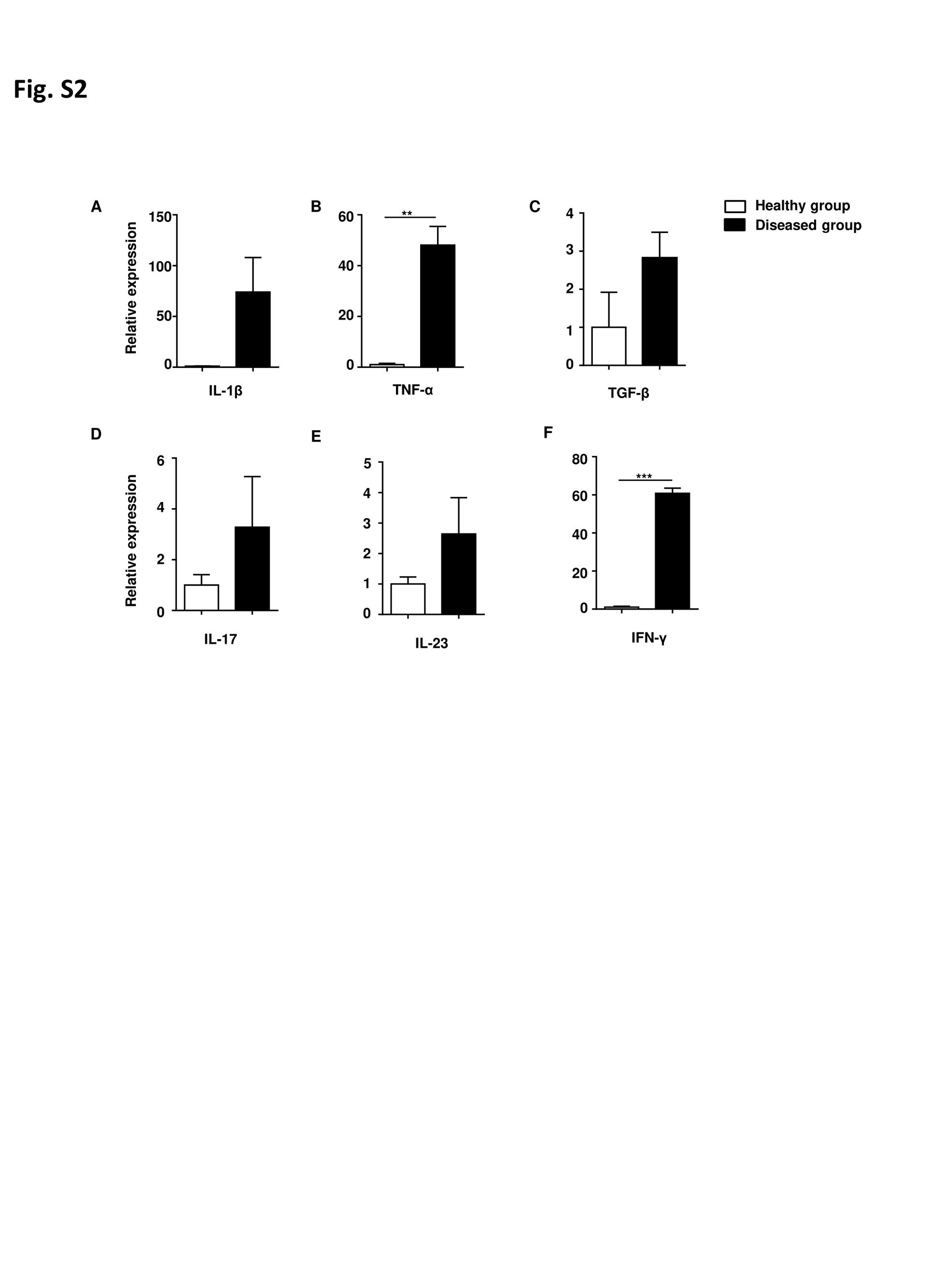

Supplement: S2 Fig — (TIF) [file pone.0299928.s002.tif]

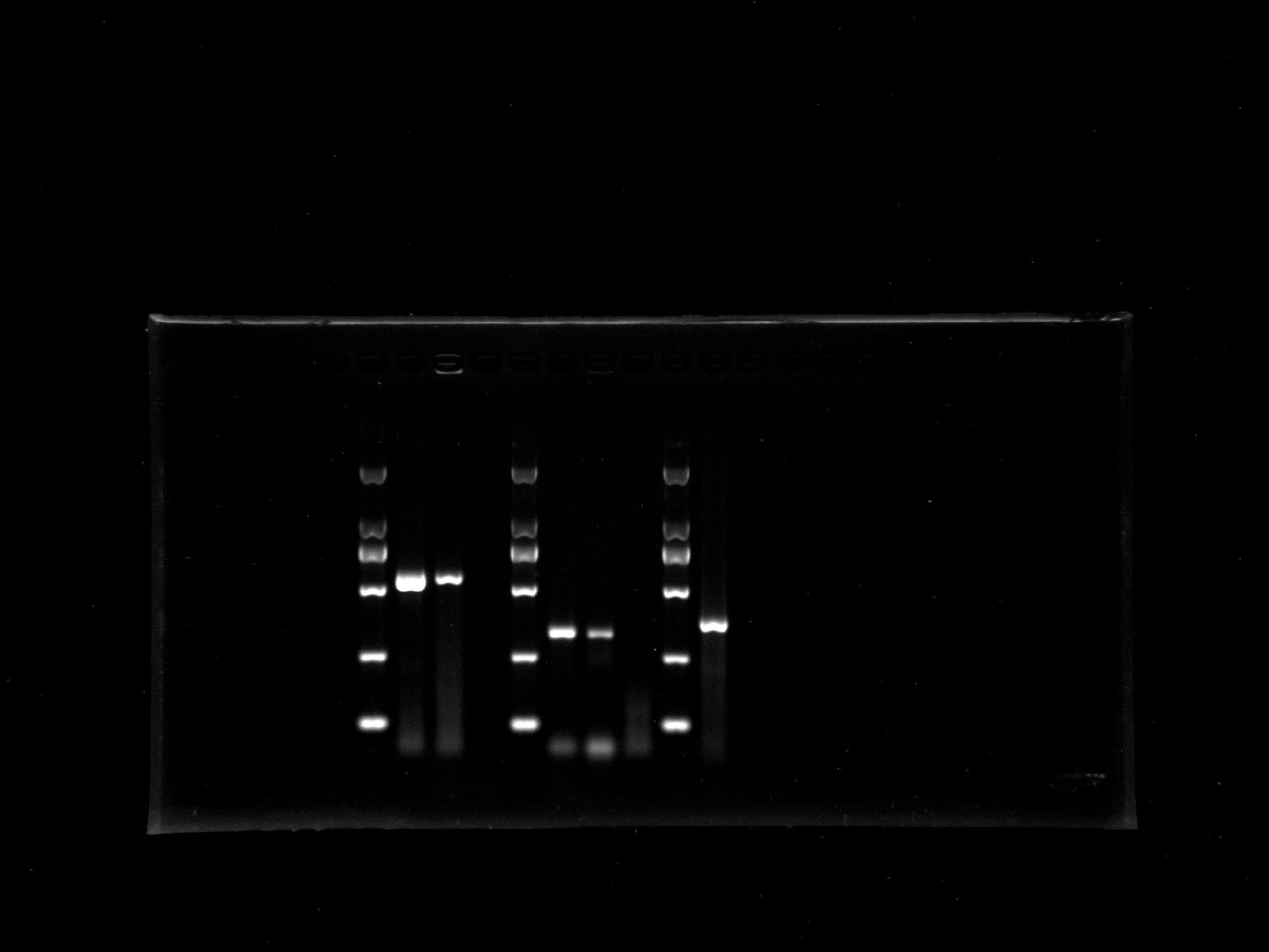

Supplement: S1 Raw images — (ZIP) [file pone.0299928.s005.zip › Original images for gels Fig 1 (A) .tif]

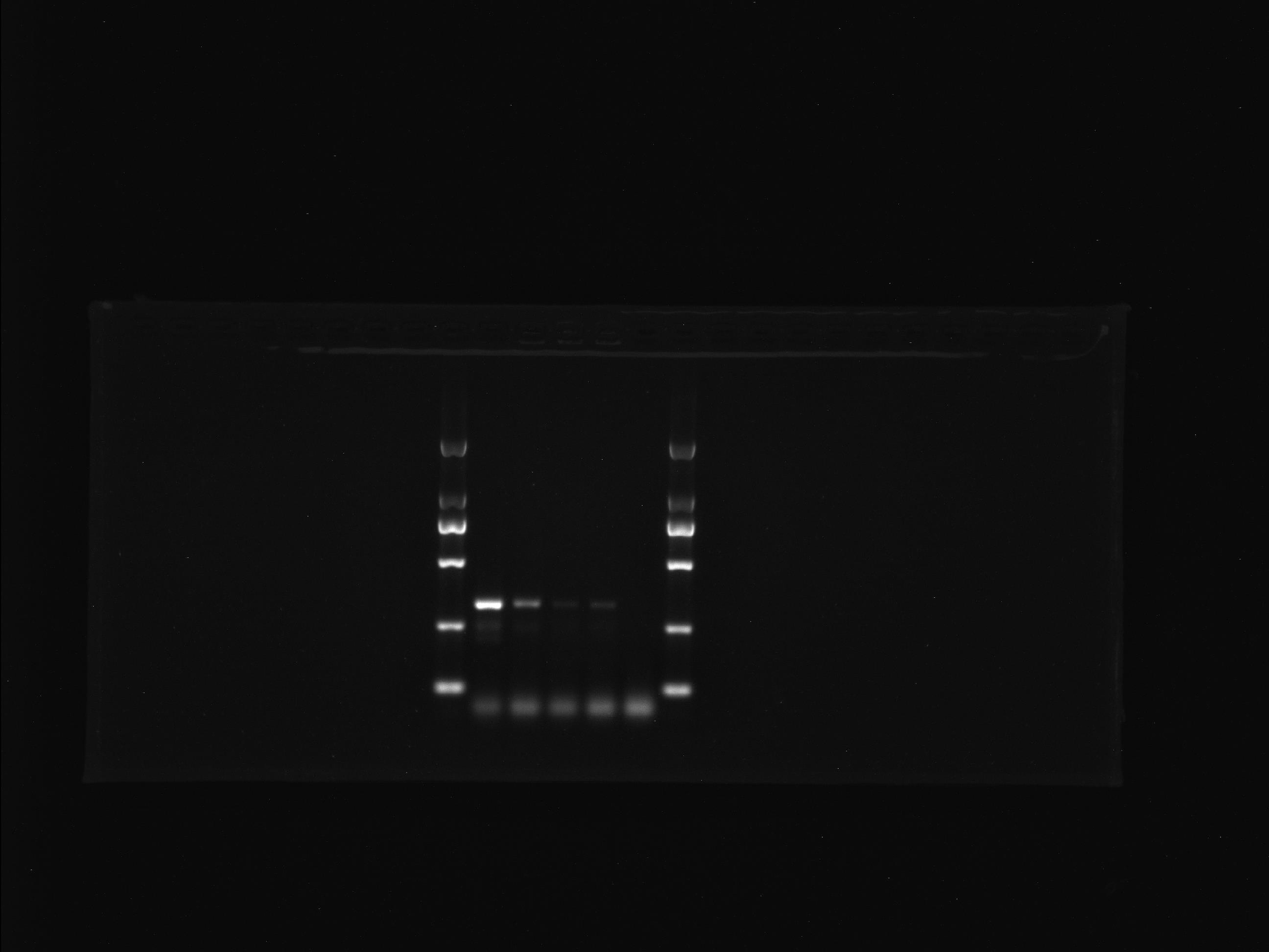

Supplement: S1 Raw images — (ZIP) [file pone.0299928.s005.zip › Original images for gels Fig 1 (B) .tif]

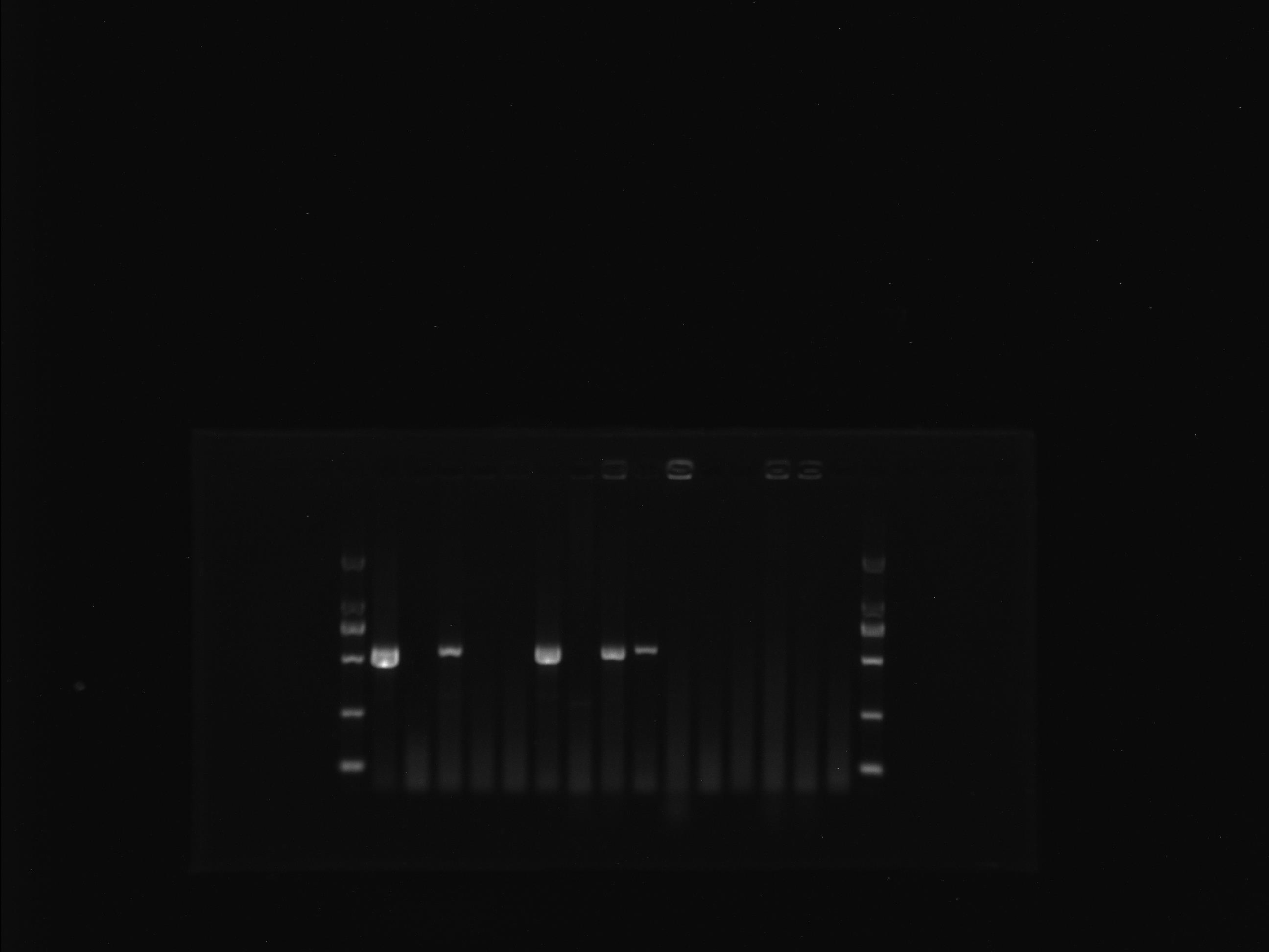

Supplement: S1 Raw images — (ZIP) [file pone.0299928.s005.zip › Original images for gels Fig 3 (A) .tif]

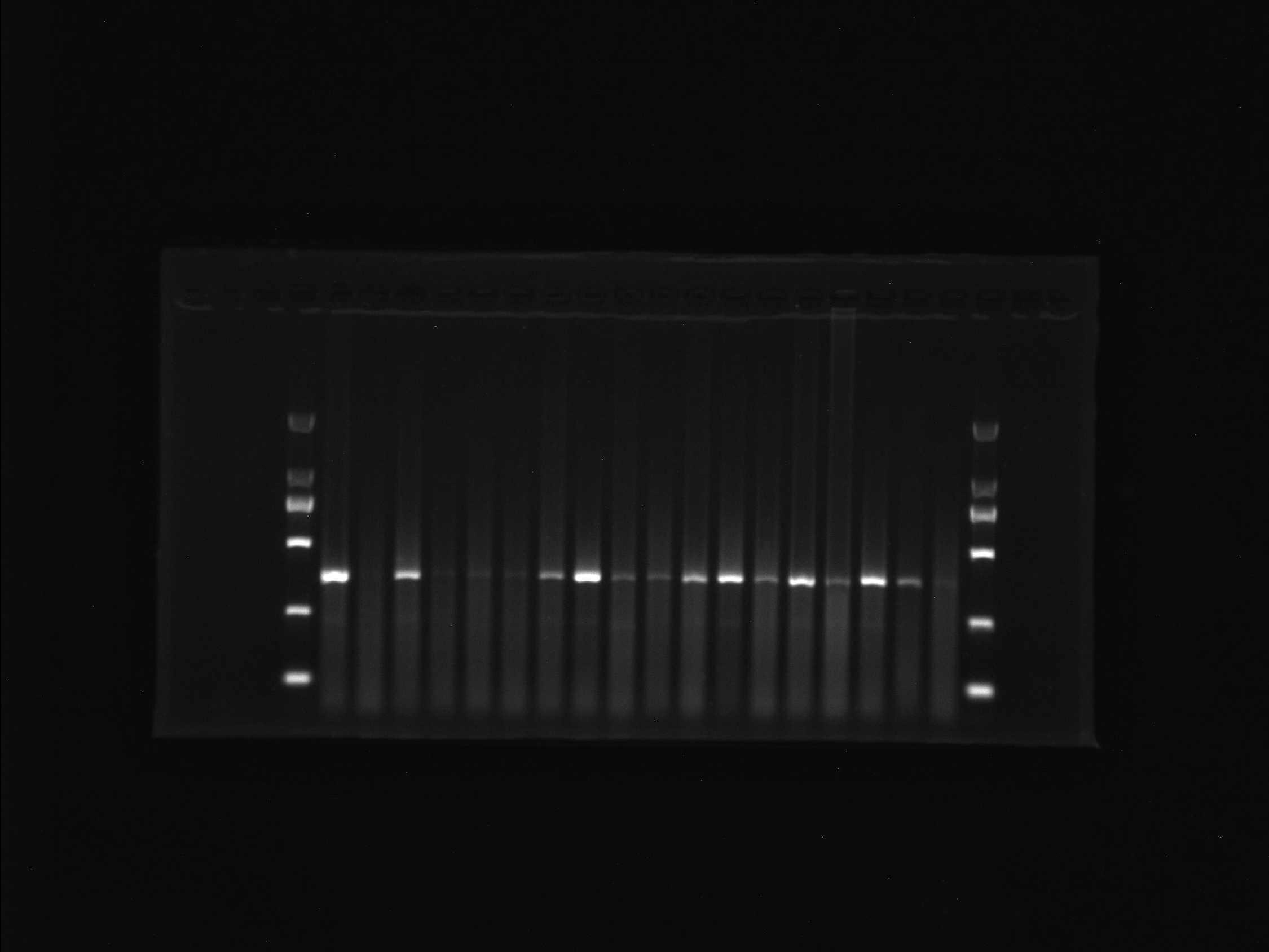

Supplement: S1 Raw images — (ZIP) [file pone.0299928.s005.zip › Original images for gels Fig 3 (B) .tif]
