# Supplementary material for: Characterization and epidemiologic analysis of mycoplasmal pneumonia of sheep in Qinghai Province
Source: PLoS One. 2024 May 21;19(5):e0299928. doi: 10.1371/journal.pone.0299928 (PMC11108190; doi:10.1371/journal.pone.0299928)
Supplement: S1 Table — (DOCX) [file pone.0299928.s003.docx]

**S1 Table**

**S1 Table. The primers used in this study for RT-PCR**

| Genes | Primer sequences (5’- sequence -3’, up: forward primers, down: reverse primers) | Gen number |
| --- | --- | --- |
| GAPDH | GCGATACTCACTCTTCTACTTTCGA | XM_005680968 |
|  | TCGTACCAGGAAATGAGCTTGAC |  |
| IL-1β | TCCACCTCCTCTCACAGGAAA | XM_013967700 |
|  | TACCCAAGGCCACAGGAATCT |  |
| TNF-α | GCACTTCGGGGTAATCGGC | NM_001286442 |
|  | GCCTTGAGGGCATTGGCAT |  |
| TGF-β | GAAGTCTAGCTCGCACAGCA | XM_005686141 |
|  | CCGAGAGAGCAACACAGGTT |  |
| IFN-γ | AGATCCAGCGCAAAGCCATA | NM_001285682 |
|  | TCTCCGGCCTCGAAAGAGAT |  |
| IL-17 | TTATCACAAGCGCTCCACCT | NM_001285725 |
|  | GCACAATGGTTCTTCCAGGTT |  |
| IL-23 | CTGAGCAGACTCCAAGCCCTAT | XM 005680346.1 |
|  | TTGGCTCACAGGTGTCTAGGTT |  |
