# Supplementary material for: Characterization and epidemiologic analysis of mycoplasmal pneumonia of sheep in Qinghai Province
Source: PLoS One. 2024 May 21;19(5):e0299928. doi: 10.1371/journal.pone.0299928 (PMC11108190; doi:10.1371/journal.pone.0299928)
Supplement: S2 Table — (DOCX) [file pone.0299928.s004.docx]

**S2 Table**

**S2 Table. Positive detection rate of Mycoplasma infection was determined by PCR.**

|  | Age(month) |  |  |  |  | Age(month) |  |  |  |  | Age(month) |  |  |  |  |
| --- | --- | --- | --- | --- | --- | --- | --- | --- | --- | --- | --- | --- | --- | --- | --- |
|  | ＜6 | mm | mm% | movi | movi% | 6-12 | mm | mm% | movi | movi% | ＞12 | mm | mm% | movi | movi% |
| Xining | 19 | 5 | 26.32% | 13 | 68.42% | 9 | 1 | 11.11% | 7 | 77.78% | 26 | 9 | 34.62% | 14 | 53.85% |
| Haidong | 29 | 6 | 20.69% | 14 | 48.28% | 28 | 7 | 25.00% | 11 | 39.29% | 13 | 1 | 7.69% | 5 | 38.46% |
| Hainan | 32 | 10 | 31.25% | 10 | 31.25% | 6 | 0 | 0.00% | 2 | 33.33% | 41 | 14 | 34.15% | 11 | 26.83% |
| Haixi | 23 | 11 | 47.83% | 5 | 21.74% | 22 | 2 | 9.09% | 9 | 40.91% | 21 | 2 | 9.52% | 6 | 28.57% |
| Haibei | 69 | 16 | 23.19% | 41 | 59.42% | 1 | 0 | 0.00% | 0 | 0.00% | 1 | 0 | 0.00% | 0 | 0.00% |
|  | Male | mm | mm% | movi | movi% | Female | mm | mm% | movi | movi% |  |  |  |  |  |
| Xining | 11 | 0 | 0.00% | 10 | 90.91% | 43 | 15 | 34.88% | 24 | 55.81% |  |  |  |  |  |
| Haidong | 35 | 8 | 22.86% | 16 | 45.71% | 35 | 6 | 17.14% | 14 | 40.00% |  |  |  |  |  |
| Hainan | 10 | 1 | 10.00% | 1 | 10.00% | 69 | 23 | 33.33% | 22 | 31.88% |  |  |  |  |  |
| Haixi | 46 | 11 | 23.91% | 11 | 23.91% | 20 | 4 | 20.00% | 9 | 45.00% |  |  |  |  |  |
| Haibei | 32 | 5 | 15.63% | 21 | 65.63% | 39 | 11 | 28.21% | 20 | 51.28% |  |  |  |  |  |
| Breed | Tibetan sheep | mm | mm% | movi | movi% | Hu Sheep | mm | mm% | movi | movi% | Crossbred Sheep | mm | mm% | movi | movi% |
| Xining | 28 | 11 | 39.29% | 12 | 42.86% | 4 | 2 | 50.00% | 3 | 75.00% | 22 | 2 | 9.09% | 19 | 86.36% |
| Haidong | 0 | 0 | 0.00% | 0 | 0.00% | 2 | 1 | 50.00% | 1 | 50.00% | 68 | 13 | 19.12% | 29 | 42.65% |
| Hainan | 25 | 8 | 32.00% | 5 | 20.00% | 43 | 11 | 25.58% | 14 | 32.56% | 11 | 5 | 45.45% | 4 | 36.36% |
| Haixi | 39 | 6 | 15.38% | 9 | 23.08% | 22 | 8 | 36.36% | 11 | 50.00% | 5 | 1 | 20.00% | 0 | 0.00% |
| Haibei | 71 | 16 | 22.54% | 41 | 57.75% | 0 | 0 | 0.00% | 0 | 0.00% | 0 | 0 | 0.00% | 0 | 0.00% |
| Feeding methods | Captive | mm | mm% | movi | movi% | Pasture | mm | mm% | movi | movi% |  |  |  |  |  |
| Xining | 48 | 13 | 27.08% | 30 | 62.50% | 6 | 2 | 33.33% | 4 | 66.67% |  |  |  |  |  |
| Haidong | 70 | 14 | 20.00% | 30 | 42.86% | 0 | 0 | 0.00% | 0 | 0.00% |  |  |  |  |  |
| Hainan | 64 | 21 | 32.81% | 23 | 35.94% | 15 | 3 | 20.00% | 0 | 0.00% |  |  |  |  |  |
| Haixi | 41 | 14 | 34.15% | 13 | 31.71% | 25 | 1 | 4.00% | 7 | 28.00% |  |  |  |  |  |
| Haibei | 50 | 8 | 16.00% | 37 | 74.00% | 21 | 8 | 38.10% | 4 | 19.05% |  |  |  |  |  |
| Feeding scale | 0-200 | mm | mm% | movi | movi% | 200-600 | mm | mm% | movi | movi% | 600-2000 | mm | mm% | movi | movi% |
| Xining | 22 | 2 | 9.09% | 19 | 86.36% | 0 | 0 | 0.00% | 0 | 0.00% | 32 | 13 | 40.63% | 15 | 46.88% |
| Haidong | 20 | 9 | 45.00% | 8 | 40.00% | 50 | 5 | 10.00% | 22 | 44.00% | 0 | 0 | 0.00% | 0 | 0.00% |
| Hainan | 37 | 13 | 35.14% | 14 | 37.84% | 0 | 0 | 0.00% | 0 | 0.00% | 42 | 11 | 26.19% | 9 | 21.43% |
| Haixi | 10 | 6 | 60.00% | 0 | 0.00% | 0 | 0 | 0.00% | 0 | 0.00% | 56 | 9 | 16.07% | 20 | 35.71% |
| Haibei | 34 | 0 | 0.00% | 29 | 85.29% | 25 | 11 | 44.00% | 8 | 32.00% | 12 | 5 | 41.67% | 4 | 33.33% |
